# Biocatalytically Oligomerized Epicatechin with Potent and Specific Anti-proliferative Activity for Human Breast Cancer Cells

**DOI:** 10.3390/molecules13112704

**Published:** 2008-11-01

**Authors:** Subhalakshmi Nagarajan, Ramaswamy Nagarajan, Susan J. Braunhut, Ferdinando Bruno, Donna McIntosh, Lynne Samuelson, Jayant Kumar

**Affiliations:** 1 Department of Chemistry, University of Massachusetts, Lowell, USA; 2 Department of Plastics Engineering, University of Massachusetts, Lowell, USA; 3 Department of Biological Sciences, University of Massachusetts, Lowell, USA; 4 U.S Army Natick Soldier Research, Development & Engineering Center, Natick, MA, 01760; 5 Department of Physics, University of Massachusetts, Lowell, USA; 6 Center for Advanced Materials, University of Massachusetts, Lowell, USA

**Keywords:** Oligomeric catechins, Enzymatic oligomerization, Anti-proliferative activity, Green chemistry, Flavonoids

## Abstract

Catechins, naturally occurring flavonoids derived from wine and green tea, are known to exhibit multiple health benefits. Epigallocatechin gallate (EGCG) is one of the most widely investigated catechins, but its efficacy in cancer therapy is still inconsistent and limited. The poor stability of EGCG has contributed to the disparity in the reported anti-cancer activity and other beneficial properties. Here we report an innovative enzymatic strategy for the oligomerization of catechins (specifically epicatechin) that yields stable, water-soluble oligomerized epicatechins with enhanced and highly specific anti-proliferative activity for human breast cancer cells. This one-pot oxidative oligomerization is carried out in ambient conditions using Horseradish Peroxidase (HRP) as a catalyst yielding water-soluble oligo(epicatechins). The oligomerized epicatechins obtained exhibit excellent growth inhibitory effects against human breast cancer cells with greater specificity towards growth-inhibiting cancer cells as opposed to normal cells, achieving a high therapeutic differential. Our studies indicate that water-soluble oligomeric epicatechins surpass EGCG in stability, selectivity and efficacy at lower doses.

## Introduction

**Figure 1:**
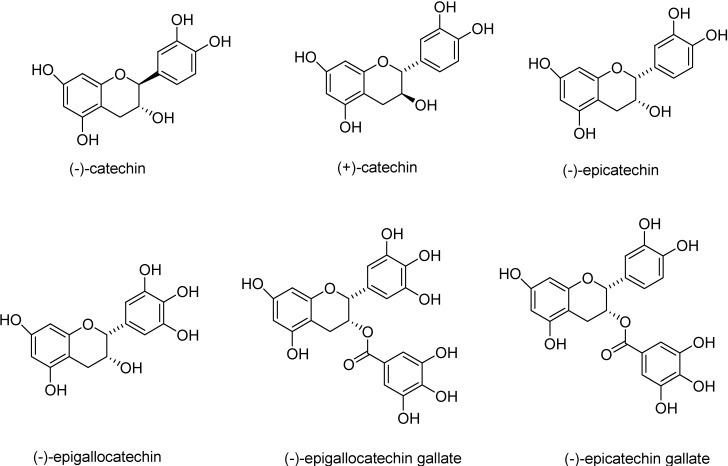
Chemical structures of the naturally occurring catechins. Among all the naturally occurring catechins, (-)-epicatechin, when oligomerized was found to possess excellent anti-proliferative activity.

Catechins, belonging to the class of flavonoids are the active agents responsible for the multiple health benefits associated with these compounds [1]. These flavonoids exhibit chemoprotective properties and are among the agents currently being tested in new, large-scale phase III clinical trials. These flavonoids have been reported to exhibit chemopreventive properties leading to reduction of incidences of skin [2], colon cancer [3] and reduce the risk of several other types of cancer (pancreas, rectum [4] and lungs [5]). They have also been found to possess anti-inflammatory, anti-allergic, anti-thrombotic and anti-viral properties. The major catechins found in green tea are (-)-epicatechin, (+)-catechin, (-)-catechin, (-)-epicatechin gallate (ECG), (-)-epigallocatechin gallate (EGCG) and (-)-epigallocatechin (EGC) (Figure 1).

EGCG, EGC and ECG are reported to be potent inhibitors of human breast cancer cell proliferation [6]. EGCG has been shown to have pleotrophic effects in its ability to inhibit tumor angiogenesis and prevent cancer metastasis by interfering with proteases, urokinase and matrix metalloproteinase (MMP) activation, as well as inhibiting MMP secretion by tumor cells. 

The impediment in the efficient use of the catechin isomers in anti-cancer applications has been their poor aqueous solubility and stability [7]. Initial results from clinical trials using EGCG for the treatment of colon, cervical cancer and skin lesions have been disappointing, with problems of poor absorption and bioconversion of the EGCG to inactive forms [8]. Moreover, their efficacy, in cancer therapy is still low as compared to commercial available anti-cancer drugs. Other reasons for the poor overall efficacy include the variability of catechin preparations (when extracted from natural sources) and the lack of standards for measuring the activity of these compounds. Nevertheless, these flavonoids provide tremendous opportunity as eco-friendly starting materials that can be chemically/enzymatically modified to yield a range of modified flavonoids with improved efficiency.

The instability of the monomeric forms of the catechins has also prompted several attempts to stabilize the monomers without loss of the therapeutic activity. In the past, polymerization has been reported as a route to increase the anti-oxidant activity [9]. However, the polymerization reactions were carried out using toxic solvents like methanol yielding water-insoluble polymers [10] which are difficult to process and not suitable for biological applications. (+)-Catechin and EGCG have been oligomerized using peroxidases [11] and laccases [12] as catalysts to yield oligomeric catechins. The multi-step synthesis of stereochemically pure oligomeric catechins has been reported but involves protection-deprotection chemistry and the use of large amount of compounds/solvents with varying levels of toxicity [13].

Enzymes promote reactions that are difficult to emulate using traditional synthetic methods. This aspect of enzyme catalysis has wider ramifications in terms of simplification of the laborious multi-step conventional chemical synthesis. Oxidoreductases such as HRP has been known to catalyze the polymerization of phenol [14] based monomers in aqueous/mixed solvent systems or in presence of biocompatible templates [15].

Here we report the enzymatic oligomerization of epicatechin as a new and eco-friendly approach to produce more stable, water-soluble oligo (epicatechins) with specific anti-tumorigenic activity. These catechins provided us with a readily available material, which lend themselves to enzymatic modification. The catechins can be extracted from green tea, making it a renewable resource as well. Extraction normally involves use of solvents [16] and/or chromatographic techniques such as reverse phase high performance liquid chromatography (RP-HPLC) [17]. Enzymes have also been used in conjunction with HPLC for extracting these catechins [18].

## Results and Discussion

### Enzymatic Oligomerization

**Figure 2:**
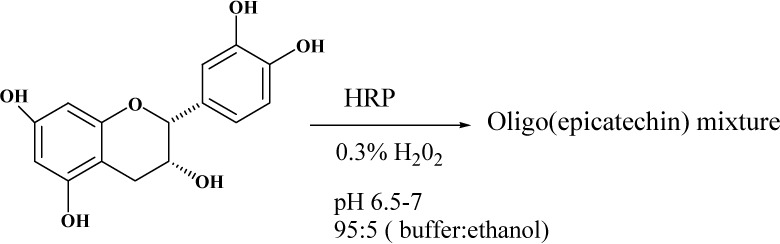
Schematic for the oligomerization of (-)-epicatechin.

In our studies, various stereoisomers of catechin [(+), (-)] and (-)-and (+)-epicatechin have been oligomerized using Horseradish Peroxidase (HRP), derived from the roots of the horseradish plant, in water-ethanol mixtures. A typical enzymatic oligomerization occurs in aqueous media buffered at pH 7 with the monomeric epicatechin, a catalytic amount of the enzyme and hydrogen peroxide, to initiate the reaction (Figure 2). 

**Figure 3:**
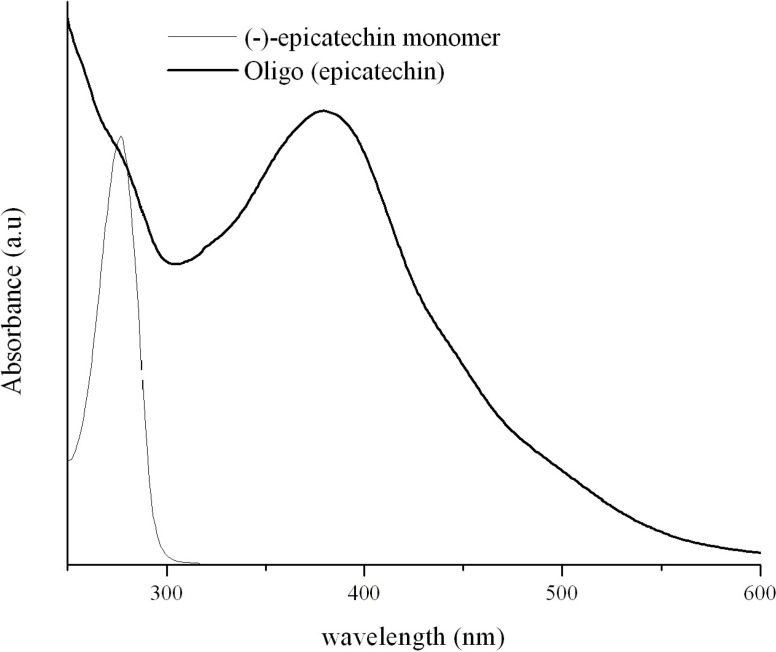
UV-Visible spectra for the oligomerization of (-)-epicatechin.

The reaction mixture was stirred overnight under ambient conditions. The resulting oligomer is separated from the monomer and lower molecular weight compounds through simple methods like dialysis and centrifugation. Figure 3 shows the UV-Visible spectra of (-)-epicatechin monomer and the resulting oligo(epicatechin) synthesized at pH 7.

As seen in the figure, the monomer shows significant absorption in the range of 250-300 nm and no absorption beyond 300 nm. The initiation of oligomerization by the addition of H_2_O_2_ leads to the appearance of a dark-red brown solution and a new broad absorption peak in the 325-550 nm range with a maxima around 390 nm. Initial MALDI-TOF studies indicate the formation of oligomers (up to 2035 a.m.u) indicating the presence of at least 7 repeat units (data not shown). 

### Circular Dichroism studies on oligomeric catechins

**Figure 4:**
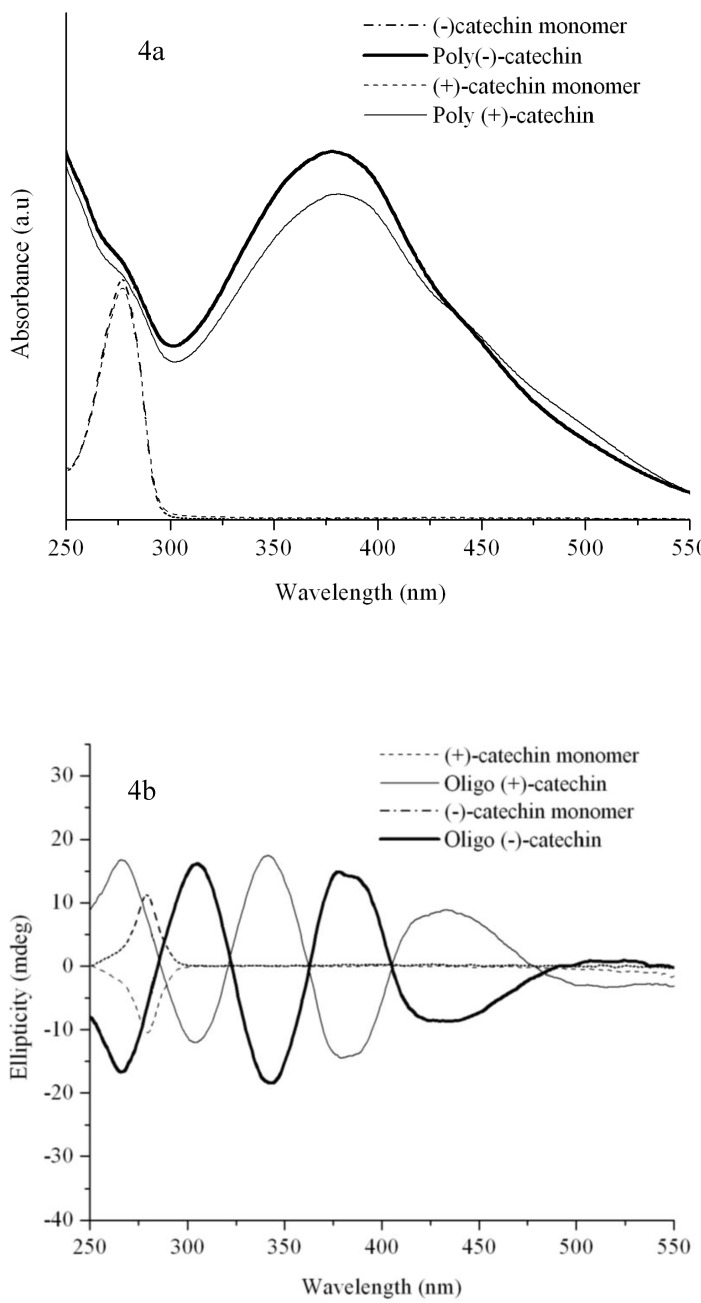
UV-Visible spectra for catechin monomers and oligomers (4b) CD spectra.

Stereoisomers of catechins have been known to exhibit characteristic Circular Dichroism (CD) [19]. CD spectroscopy can also be used to study the stereochemistry and/or the secondary structure of the oligomers. The CD spectra of oligomeric forms of (+) and (-) catechins indicate that the oligocatechins adopt unique secondary structures that are exact mirror images of each other (Figure 4b).

The oligomer of (-)-epicatechin also exhibits a unique CD spectrum [20]. Further studies are underway to deconvolute and carry out specific peak- assignment. We hypothesize that oligocatechins with distinct secondary structures can be capable of mimicking specific ligand(s), accessing growth control pathways with the possibility for enhancing the therapeutic activity.

### In-vitro studies on the anti-proliferative activity of oligomerized epicatechins

**Figure 5:**
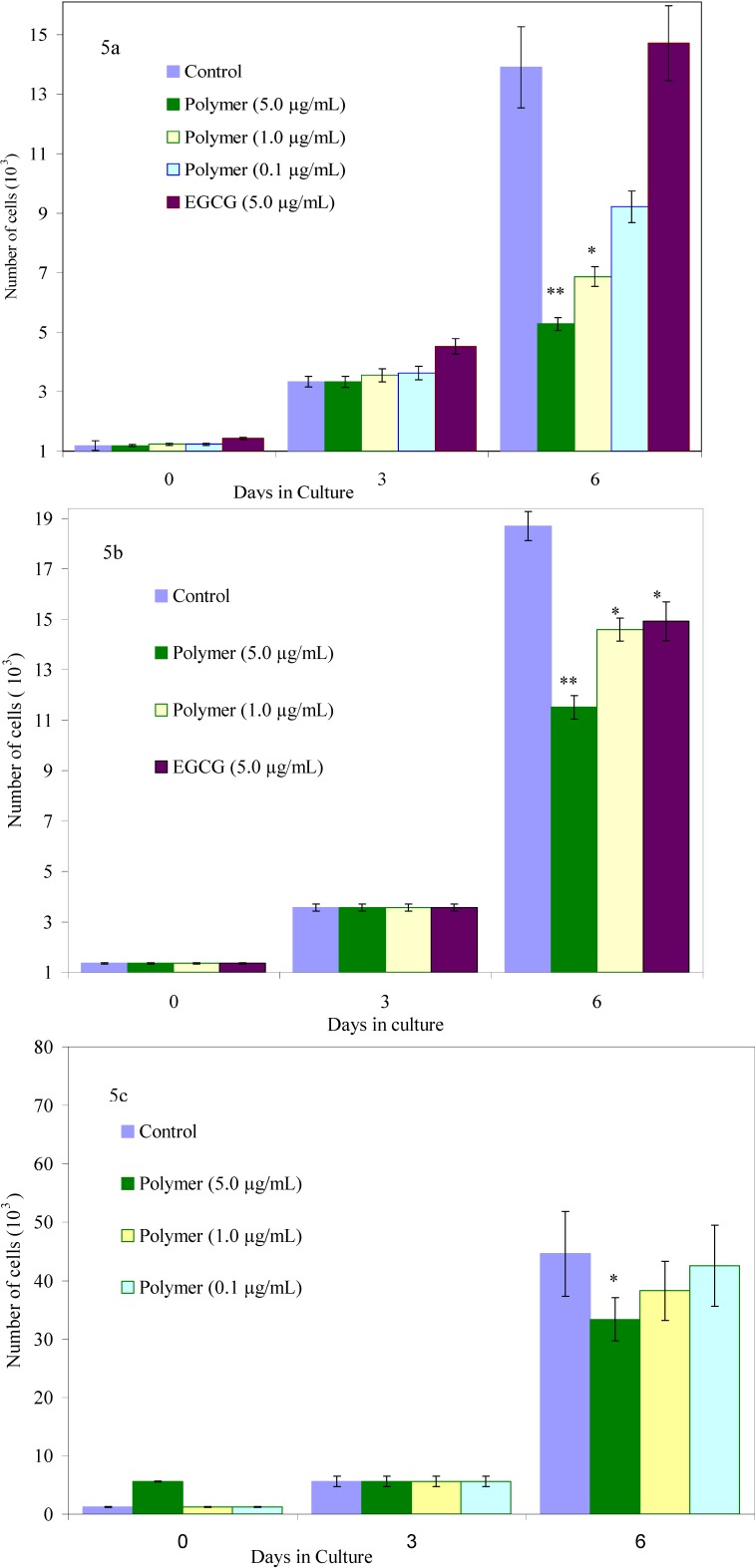
Effects of oligo(epicatechin) on the growth of (5a) low metastatic human breast cancer cells (5b) high metastatic human breast cancer cells (5c) normal cells [p values <0.05(*) or <0.001(**)].

**Figure 6:**
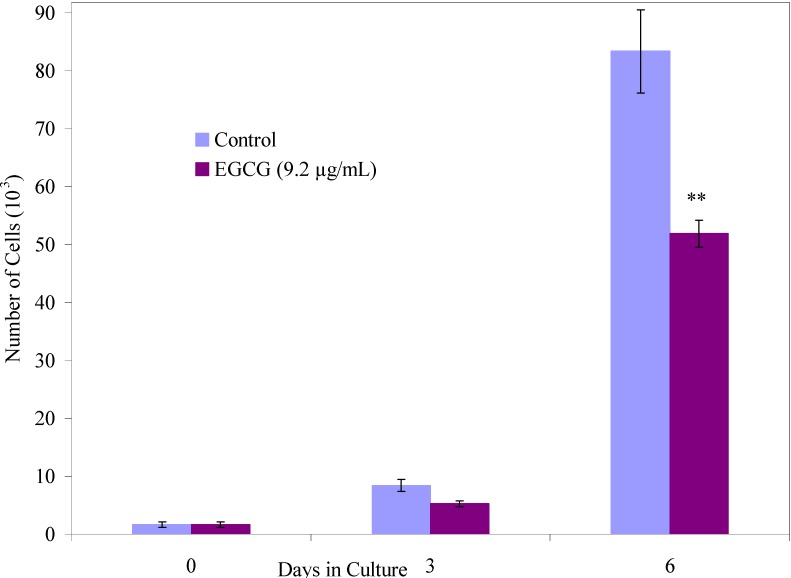
Effect of EGCG on the growth of normal cells.

We have conducted proliferation studies on normal and malignant human breast cancer cells using this new-class of oligoepicatechins. A series of human cancer cell lines (high and low metastatic breast cancer cells, colorectal cancer, and nasopharyngeal cancer) were selected and the efficacy of these oligomeric compounds in inhibiting the growth of these human cancer cells was then analyzed in a dose response study. The experimental means were compared to the means of untreated cells harvested in parallel and the data was pooled for replicate experiments. Among all oligomerized forms of the catechins, oligo(epicatechin) in ethanol (Oligo EC/EtOH) proved to be the most efficient in growth inhibiting the cancer cells without effecting normal cell growth at low doses. At effective doses in which the oligo(EC/EtOH) inhibits the growth of cancer cells, the monomer and HRP did not show any activity. The oligomer was tested over a dose range of 0.1 to 5 μg/ml in parallel with EGCG tested at 5 μg/mL and 9.2 μg/mL. These doses of EGCG were selected based on the literature showing EGCG is not effective at inhibiting human breast cancer cells *in-vitro* at doses below 5 µg/mL [21]. In our studies, we also found that EGCG at 5 μg/mL was a poor inhibitor of both the breast cancer cell lines (Figure 5a, Figure 5b) and it also growth inhibited normal mammary epithelial cells to the same extent as the cancer cells (Figure 6).

Though there have been numerous reports on the ability of EGCG to inhibit cancer cell growth, the inhibitory activity requires a very high concentration of EGCG (>20 μM). While these concentrations are achievable *in-vitro*, they may not be achievable *in-vivo* [22]. EGCG failed to inhibit MCF-7 cells (low metastatic breast adenocarcinoma) (Figure 5a) and only inhibited the high metastatic breast cancer cells by 22% (p<0.05) (Figure 5b) at a 5 µg/mL dose when tested in parallel. This is consistent with previously published results [22]. EGCG also growth inhibited MCF-12A cells (normal mammary epithelial cells) exhibiting no selectivity for cancer cells. EGCG is known to inhibit MCF-7 cells by 38% and MDA-MB-231 cells (high metastatic breast adenocarcinoma) by 34% over a seven day treatment when used at a dose of 9.2 µg/mL. Lower doses were found to be ineffective [23]. Our results therefore indicate that EGCG does not exhibit any therapeutic differential in growth inhibition of cells. In striking contrast, the growth of MCF-12A cells was not significantly affected when treated with our new oligomer namely oligo(EC/EtOH) at most doses (Figure 5c). At the same doses, MCF-7 cells were 75% growth inhibited (Figure 4a) whereas MDA-MB-231 cells show a dose response at 1.0 and 5.0 μg/mL (Figure 5b).

Moreover, the oligoepicatechin we have synthesized enzymatically outperforms and is more selective than EGCG. Furthermore, the oligocatechin exhibits a specific inhibitory effect for cancer cells, is soluble in aqueous solutions and very stable. This new oligomeric compound also retained complete activity after being stored in solution for 3 months. It is worthwhile to point out that EGCG was found to degrade completely by 3 hours when incubated in buffered solutions.

Different strategies have been adopted for the synthesis of oligomeric catechins [24]. Kozikowski *et al.* have reported the chemical synthesis of epicatechin oligomers and the ability of these oligomers to growth inhibit human breast cancer cell lines (MDA MB 231) [13]. But the dose required for growth inhibition (100 μg/mL) is at least 20 times higher than those observed with our enzymatically synthesized oligomeric epicatechins (>75% inhibition even at 5 µg/mL dose). Recently oligonol, a compound containing catechin-type monomers and oligomeric proanthocyanidins has also been shown to be effective in growth inhibiting MCF-7 and MDA-MB-231 cells at 20 μg/mL [25].

In addition, Oligo(EC/EtOH) was also found to be effective in inhibiting the growth of human colorectal and naso-pharyngeal human cancer cell lines at similar low doses (data not shown). Studies are currently underway to identify the mechanism of growth inhibition of human cancer cells by oligo(epicatechins). It does not appear to be through a mechanism of inducing apoptosis but does achieve a cell cycle arrest in the G2 part of the cell cycle. 

Initial NMR studies on the oligomeric mixture were not conclusive. Peroxidase-catalyzed polymerization of phenolic compounds is carried out in the presence of H_2_O_2_, which acts as an oxidant, and the peroxidase cycle involves a two-electron oxidation step and two one-electron reduction steps. The free radicals formed undergo oxidative coupling to produce dimers and other oligomeric/polymeric products. HRP catalyzed oligomerization reactions are hence quite complex, since the radicals formed can couple in numerous ways. There have been considerable efforts in understanding the nature of coupling in the HRP catalyzed modification of substituted phenols [26] and other stereoisomers of catechin [27]. The reactions have been quenched and even at initial stages of the reaction, multiple products have been isolated and identified using 1D and 2D NMR.

In this research work, we have identified oligo(epicatechin) to be the most potent oligomer from among the family of catechins in inhibiting growth of human breast cancer cells. High Performance Liquid Chromatography (HPLC) studies indicate the oligomer to be a mixture comprising of three different fractions. Based on absorption of the fractions at 210 nm, the lower molecular weight compounds are present to the extent of around 30%. HPLC studies on the HRP catalyzed oligomerization of (+)-catechin has been reported to result in the formation of oligomeric(catechins) as well [28]. At the current time, separation of these oligomeric epicatechin fractions and testing of anti-proliferative activity (both in vitro and in vivo) are currently being pursued. We plan to investigate the structure of the most potent fraction *in-vitro* and *in-vivo*. The nature of the coupling and the mechanism of the reaction are under investigation.

## Conclusion

In conclusion, we have developed a new class of soluble and biocompatible oligomeric epicatechins and demonstrated their effectiveness in inhibiting the growth of human cancer cells. The unique enzymatic synthetic protocol augments the stability, biological activity and compatibility of these epicatechins with biological systems. These oligo (epicatechins) are highly stable when compared to EGCG and retain their activity for more than 3 months. These oligomers also exhibit enhanced growth inhibitory activity for cancer cells, and do not affect the growth of normal human mammary cells at the same dose range, achieving a high therapeutic ratio. The entire synthetic protocol used here is environmentally benign and does not need any multi-step protection/de-protection processes or the use of carcinogenic materials at any point. The raw materials are obtained from renewable sources and are readily available. Preliminary studies also indicate that the oligo(epicatechins) are effective even without elaborate purification procedures.

## Experimental

### General

Horseradish peroxidase (HRP, EC 1.11.1.7) type II, 150-200 units/mg solid and all catechin monomers was purchased from Sigma chemicals Co. (St. Louis, MO). Hydrogen peroxide (30 wt%) were purchased from Aldrich Chemicals Inc., Milwaukee, WI and was diluted with water to make 0.3% H_2_O_2_ solution. All other chemicals were purchased from Aldrich and were of reagent grade or better. UV-Visible spectra were obtained using a Perkin-Elmer Lambda 9 UV-Visible-near-IR spectrophotometer. Circular Dichroism (CD) spectra were obtained using a Jasco CD spectrometer J-720. 

*Synthesis of oligo(epicatechin):* The oligomerization reactions were carried out in 95:5 (v/v) mixtures of phosphate buffer (pH 7) and ethanol. For a typical reaction, (-)-epicatechin (3.44 mM) was dissolved in ethanol (500 μL) and then phosphate buffer (8 mL) was added to the above solution. The pH was adjusted to be between 6-6.5. A solution of HRP (4 mg in 0.5 mL deionized water) was to the reaction mixture and the reaction was initiated by the dropwise addition of 0.3% H_2_O_2_ (1500 μL). The reaction was allowed to proceed for 24 hours. The product formed was dialyzed to remove unreacted monomers and oligomers. The oligomer was then dried under vacuum for 72 hours. The gravimetric yield was between 65-70%. A similar procedure was followed for the oligomerization of other stereoisomers of catechin.

*Proliferation assays:* Cells (1,000 per well) were plated into 48-well assay plates and proliferation studies were performed using MCF-7 (HTB-22, low metastatic breast adenocarcinoma), MDA-MB-231 (HTB-26, high metastatic breast adenocarcinoma) and MCF-12A [CRL#10782, normal mammary epithelial cells obtained from the American Type Culture Collection (ATCC, Manassas, VA)]. Stock cultures of MCF-7 cells were maintained in MEM with 10% Fetal Calf Serum, 1% fungizone, 1% GPS, NEAA, Sodium pyruvate and bovine insulin. GPS solutions contained 200 mM glutamine, 10K U penicillin, 10 mg/mL streptomycin solution. A stock solution of insulin was prepared (10 mg/mL) and was used at 10 µL/mL. MDA-MB-231 cells were in DMEM with 10% Calf Serum, 1% fungizone and 1% GPS. MCF-12A cells were in DMEM/ F-12 with 10% Horse Serum, 20 ng/ml hEGF, 100 ng/ml cholera toxin, 500 ng/ml hydrocortisone and bovine insulin in DMEM with 10% calf serum containing glutamine and penicillin streptomycin, according to the manufacturers instructions. Colorectal cancer cells HT29 (ATCC, HTB-38) are maintained in McCoy’s 5A medium with 10% fetal bovine serum, 1% fungizone and 1% glutamine, penicillin, streptomycin solution. Nasopharyngeal cancer cells FaDu (ATCC, HTB-43, pharynx squamous cell carcinoma) are maintained in MEM with 10% fetal bovine serum, NEAA, Fgz, and 100 mM sodium pyruvate (diluted 1:100 for cell culture). All these cell lines are maintained in a humidified incubator at 37°C, with 5% CO_2_.

T_0_ counts were taken at 24 hours after plating, and cells were refed with growth media, with or without vehicle alone, EGCG or oligo(catechins), at various doses. Every three days, in triplicate, per treatment per time point, wells were harvested using trypsinization, and cell numbers determined electronically using a Coulter counter. Remaining wells were re-fed growth media, with or without treatments. Experiments were performed a minimum of three times and only experiments were included where control cells exhibited a minimum of 2.5 cell population doubling over the first 6 day period. The experimental means are compared to the means of untreated cells harvested in parallel and the data is pooled for replicate experiments, standard deviations and statistical differences calculated using student T-tests (p values <0.05(*) or <0.001(**) are considered significant).
